# 3D Bioprinting for Biomedical Applications

**DOI:** 10.34133/bmef.0010

**Published:** 2023-02-15

**Authors:** Bin Kong, Yuanjin Zhao

**Affiliations:** ^1^ Department of Rheumatology and Immunology, Institute of Translational Medicine, The Affiliated Drum Tower Hospital of Nanjing University Medical School, Nanjing 210002, China.; ^2^ Oujiang Laboratory (Zhejiang Lab for Regenerative Medicine, Vision and Brain Health), Wenzhou Institute, University of Chinese Academy of Sciences, Wenzhou, Zhejiang 325001, China.; ^3^ Chemistry and Biomedicine Innovation Center, Nanjing University, Nanjing 210023, China.

**Fig. 1.**
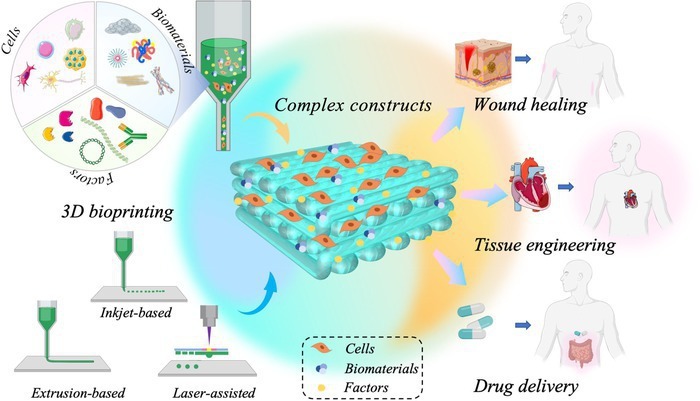
The schematic illustration of 3D bioprinting and its application in the biomedical fields.

Three-dimensional (3D) printing, referring to a type of additive manufacturing, has emerged as a promising fabrication technique in the past decades since it can create 3D objects with desired architecture by precise control over the deposition of successive layers of various materials. Benefiting from these advantages, 3D printing has been extensively applied in varied areas of science and engineering. With the rapid development and comprehensive investigation of 3D printing technology, 3D bioprinting begins to emerge and paves the path for new design possibilities in the field of tissue engineering and regenerative medicine. A relevant development is using 3D printing to create biomedical devices, such as stents and splints for clinical use [1,2]. 3D bioprinting can engineer 3D constructs in vitro through precise deposition and assembly of biomaterials, functional biochemicals, and living cells. The spatiotemporal regulation of this strategy over the communications between individual cells and cell-extracellular matrix (ECM) imparts the designed construct with the ability to simulate the structures and functions of native tissues or organs. Based on 3D bioprinting, researchers have demonstrated the fabrication of functional objects with mechanical and biological features appropriate for the functional recovery of tissues or organs [3] (Fig. 1).

The biological materials used in 3D bioprinting are usually derived from natural or synthetic materials, which can be static or dynamic. For instance, dynamic materials can be biodegraded by hydrolysis or enzymatic digestion. Biomaterials with advanced and dynamic functions can be synthesized through chemical methods, which should be endowed with ﻿a proper crosslinking mechanism to facilitate bioprinter deposition, good biocompatibility, and structural and mechanical long-term stability [4]. The benefits of natural polymers used in 3D bioprinting are their resemblance to the native ECM and intrinsic bioactivities. For synthetic polymers, they can be endowed with particular physiochemical abilities to accommodate a specific application. However, the disadvantages of synthetic polymers remain challenging, such as inferior biocompatibility and mechanical property reduction during the degradative process. Nevertheless, a synthetic hydrogel with hydrophilic and absorptive properties is appealing for 3D bioprinting applied in biomedical fields because of the easy control of its physiochemical abilities during the synthetic process. High-throughput screening of different material variations allows quick engineering of materials with unique formulations, and a variety of biomaterials are utilized as cellular constructs and mimetic environments in tissue engineering [5]. Materials used for 3D bioprinting have to satisfy particular standards determined by the respective technique, such as extrusion- or inkjet-based bioprinting and laser-assisted bioprinting. In addition, the spatial resolutions of printed constructs vary from the nanoscale to the milliscale, depending on the bioprinting technologies as well. Extrusion-based 3D bioprinting was one of the first verified bioprinting paradigms. In this strategy, biological materials, which are termed bioinks, are loaded into the print head and extruded from the nozzle in a manner of layer-by-layer to produce fine filaments and build desired constructs. Due to the various compatibility of the materials, ease of handling, moderate printing environment, and cost-effectiveness, extrusion-based 3D bioprinting has become the most widely used bioprinting approach in the biomedical fields [6].

Over the past decades, 3D bioprinting has been developed for wound healing and skin regeneration. Notably, the use of in situ 3D printing on the wound area can offer a number of benefits. It allows the straightforward use of the cultured cells on the injured site and the fabrication of customized scaffolds that can well match the shape of the injured area to facilitate the maturation and regeneration of the skin. Besides, 3D bioprinting also exhibits considerable potential in tissue engineering, which can offer bioengineering scientists an unforeseen ability to construct complicated 3D biological structures. Substantial advances have been implemented in the development of robust biomanufacturing systems to fulfill the diverse demands of bioprinting complex bionic structures with various cells and materials. Nevertheless, major technical challenges remain in successfully exploiting functional alternatives of tissues or organs. In addition, tailored drug delivery and personalized drug manufacturing methods have been assumed via the use of 3D bioprinting. These methods are designed to produce customized prescriptions on demand to fulfill patient-specific pharmacogenomic, anatomical, and physiological needs. By far, the broadest range of bioprinting processes used to manufacture personalized drugs is inkjet-based bioprinting.

**Fig. 2.**
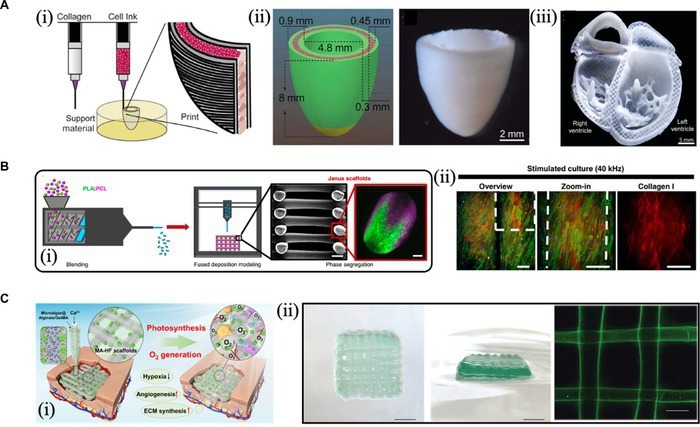
(A) (i) The schematic illustration of ﻿dual-material printing based on a collagen ink and a cell ink. (ii) ﻿Model and ﻿micrograph of a printed ventricle with cardiac cells, collagen shells, and a collagen-only section. (iii) ﻿Side and top views of the printed ventricle stained with calcium-sensitive dye and respective calcium mapping. (iv) ﻿Cross-sectional view of the collagen heart and high-fidelity image of the trabeculae in the left ventricle and the septal wall between ventricles. Reproduced with permission from [8]. Copyright 2019 The authors. (B) ﻿(i) Schematic diagram of the process to create phase-segregated scaffolds. (ii) Collagen deposition of bone mesenchymal stem cells cultured for 21 days on Janus scaffolds under dynamic conditions. Reproduced with permission from [9]. Copyright 2021 The authors. (C) (i) Illustration of in situ 3D bioprinting living photosynthetic scaffolds for autotrophic wound healing. (ii) Microfluidic bioprinting process and different views of the printed hollow fibrous scaffolds. Reproduced with permission from [10]. Copyright 2022 The authors.

The ability of 3D bioprinting is being developed at a level where a wide range of materials and cell types are molded into the constructs that approach clinically related dimensions and microstructures [7]. The bioprinted tissues, such as skin, heart, cartilage, and blood vessel, illustrate the possibility of this technology in the biomedical fields. For instance, the dual-material printing strategy was used to print a left ventricular model based on cardiomyocytes and collagen (Fig. 2A). The authors analyzed the function of the model and observed the electrophysiological behavior and ventricular contraction phenomena associated with heart rate disorders [8]. Camarero-Espinosa and Moroni [9] printed a dynamic double-sided scaffold that could activate nanovibrations with external ultrasound and deliver them to the surrounding cells to modulate functional cell expression for bone regeneration (Fig. 2B). In addition to the tissue-engineered scaffolds, customized drug delivery methods and the production of individualized drugs have been assumed by the application of 3D bioprinting technology. These methods are intended to produce customized prescriptions on demand to satisfy patient-specific pharmacogenomic, anatomical, and physiological needs. Besides, 3D bioprinting has exhibited potential in wound healing. Our recent work enabled the in situ printing of microalgae scaffold materials on skin wound sites through 3D bioprinting (Fig. 2C). This living scaffold with photo-controlled oxygen production could continuously supply oxygen to hypoxic wounds and promote vascular regeneration, thus significantly improving would healing [10].

Although 3D bioprinting has been developed at a rapid pace in the biomedical fields worldwide, this strategy still faces significant hurdles. The fabrication of tissues or organs with integral functions still needs to be within reach. On the one hand, the construction of vascular and neural networks is still the main challenge and critical milestone for the fabrication of functional constructs. Several approaches have been investigated to achieve vascularization, but only minor advancements have been reported in innervation engineering [7,11,12]. On the other hand, the immune response mechanism within the tissues or organs needed to be fully enlightened to allow the functional transplantation of biologically manufactured constructs after implantation. Specifically, the communications between individual cells and cell-ECM should be well understood to recreate the complicated microstructure of ECM and multiple cell types, thus recapitulating organs with anatomy and physiology. In addition, the printing resolution and fabrication speed should be improved to construct large-scale tissue or organs using a variety of biocompatible biomaterials for the clinical transition. Most reported studies have demonstrated the printing of much smaller constructs than native tissues or organs. Although they have exhibited high cell viability and expressed specific functions, these constructs remain at the laboratory research stage and are still far from clinical use. Negotiating the bioprinting resolution and the biocompatibility of bioprinting materials remains challenging. Advances in the software developing to control the 3D bioprinting system have resulted in the execution of scripts to fabricate complicated porous network structures and multi-material deposits. Nevertheless, most manufactured biological constructs are as yet featured by simple structures that are not commensurate with the intricacy of native tissue. The manufacturing speed has to be improved to create the clinically related size of a construct. An approach to reach this goal is to produce miniature functional tissue blocks, which can be extended to clinically related sizes by utilizing macro-scale scaffolds to connect the tissue blocks.

Besides, substantial challenges also exist in scaling up and commercializing the bioprinted implant with varied functionalities, for example, built-in computational process, disease monitoring, and tissue or organ repair. In practical applications, the structural design, cellular source, and manufacturing logistics should be highly customized to meet the individual need of each patient. Thus, how to enhance the cost-effectiveness of the customized bioprinted constructs needs to be solved in the future. Although 3D bioprinting has been widely used in laboratories and companies, there is no uniform standard for this strategy in terms of the printing material and process. Standardizing the material of bioprinting to a certain degree could have a favorable influence on the development time of the product. Meanwhile, standardizing the material and process of bioprinting could also facilitate clinical translation of the printed construct and optimization of the process, minimizing the resource needed to develop the underlying material and technical procedure. Notably, the computer controls the bioprinting process. Therefore, the assembly of 3D bioprinting with artificial intelligence would achieve an intelligent, automated, responsive, and predictable printing process. The computational design in 3D printing may become major development orient in the future. Overall, 3D bioprinting is a promising strategy and powerful tool in the biomedical fields, and we look forward to more substantial and encouraging accomplishments in 3D bioprinting through further efforts.
